# Routine antibiotics may not be needed to treat uncomplicated right diverticulitis: A retrospective cohort study

**DOI:** 10.1371/journal.pone.0255384

**Published:** 2021-07-29

**Authors:** Kil-yong Lee, Jaeim Lee, Youn Young Park, Seong Taek Oh

**Affiliations:** Division of Coloproctology, Department of Surgery, College of Medicine, Uijeongbu St. Mary’s Hospital, The Catholic University of Korea, Seoul, South Korea; Ohio State University Wexner Medical Center Department of Surgery, UNITED STATES

## Abstract

We aimed to investigate whether antibiotic administration is necessary for patients with uncomplicated right colonic diverticulitis. Data from patients diagnosed with uncomplicated right colonic diverticulitis, who received inpatient treatment at a single center between January 2019 and January 2021, were retrospectively examined. The patients were divided into two groups according to whether antibiotics were administered. The patients were matched between groups using propensity score matching in a 1:1 ratio using logistic regression with the nearest-neighbor method. The primary study outcome was the length of hospital stay, and the secondary outcomes were time to the introduction of sips of water and a soft diet. The study included 138 patients who received antibiotics and 59 who did not. After propensity score matching, 55 patients were assigned to each treatment group. There was no significant difference between the two groups in terms of age (p = 0.772), sex (p>0.999), body mass index (p = 0.121), prehospital symptom duration (p = 0.727), initial body temperature (p = 0.661), white blood cell count (p = 0.688), or C-reactive protein level (p = 0.337). There was also no statistically significant difference in the length of hospital stay between the no antibiotic and antibiotic groups (3.1±0.7 days vs. 3.0±1.0 days, p = 0.584). Additionally, no significant difference was observed between the no antibiotic and antibiotic groups with respect to time to sips of water (2.1±0.7 days vs. 1.8±0.9 days, p = 0.100) and time to the introduction of a soft diet (2.4±0.8 days vs. 2.1±0.9 days, p = 0.125). The findings suggest that routine antibiotics may be not required for treating patients with uncomplicated right colonic diverticulitis.

## Introduction

The World Society of Emergency Surgery (WSES) guidelines for patients with uncomplicated left colonic diverticulitis published in 2020, state that antibiotics do not need to be prescribed for immunocompetent patients in the absence of systemic inflammation [1]. The guidelines also recommend that the diagnosis and treatment of uncomplicated right colonic diverticulitis should be performed similarly to those of left colonic diverticulitis [2]. However, there have been few studies regarding antibiotic use specifically for uncomplicated right colonic diverticulitis, which occurs at a young age and is associated with fewer complications [2–4].

The recommendations of the WSES guidelines for the prescription of antibiotics remain debatable owing to the lack of evidence regarding antibiotic therapy for uncomplicated right colonic diverticulitis. According to guidelines published by the Japan Gastroenterological Association in 2019, antibiotics are recommended for patients with uncomplicated right colonic diverticulitis, because there are not enough studies to justify discontinuing its use [5]. However, recently published results of a randomized control trial (RCT) in patients with uncomplicated right colonic diverticulitis suggest that there is no need for antibiotics in these patients [6].

Since these findings are from a single study, more evidence is needed to support a change in recommendations. Therefore, our study aimed to investigate the need for antibiotics in patients with uncomplicated diverticulitis of the right colon.

## Materials and methods

This study was approved by the institutional review board (IRB) of the Catholic University of Korea (IRB number: UC21RISI0024), and was performed in accordance with the relevant guidelines and regulations of the IRB. The investigation conformed with the principles outlined in the Declaration of Helsinki of 1964. Informed consent for participation was waived under IRB approval from the institutional review board of the Catholic University of Korea.

### Patients

Patients who visited Uijeongbu St. Mary’s Hospital for suspected right colonic diverticulitis between January 1, 2019 and January 15, 2021, were included in this study. We retrospectively reviewed data from the hospital database for patients who were admitted to the hospital with right colonic diverticulitis since January 2019.

Right colonic diverticulitis was diagnosed using computed tomography (CT). Acute uncomplicated diverticulitis was defined according to previous studies [7, 8], as the presence of colonic diverticular disease with localized colonic wall thickening and/or stranding of the pericolonic fat. CT images showing the appearances of complicated diverticulitis (defined as the presence of pericolonic or abdominal abscesses, localized or free extraluminal gas, or contrast, obstruction, or fistula formation) or the presence of an associated mass lesion were excluded.

The inclusion criteria were as follows: diagnosis of (1) primary right colonic diverticulitis (cecum, ascending colon, and transverse colon) and (2) uncomplicated diverticulitis. The exclusion criteria were as follows: (1) recurrent diverticulitis, (2) complicated diverticulitis, (3) confusion with acute appendicitis, (4) refusal of treatment and subsequent discharge from the hospital, and (5) death during hospitalization for reasons not related to diverticulitis. Patients were divided into two treatment groups: those who received antibiotics and those who did not.

### Variables

The included variables were demographics and clinical information at the time of admission, such as age, sex, height, body mass index (BMI), body temperature, history of smoking and alcohol use, past medical history (e.g., history of hypertension, diabetes, and/or cardiac disease), laboratory findings (e.g., white blood cell [WBC] counts and C-reactive protein [CRP] levels), time to introduction of sips of water and a soft diet, hospital stay duration, 30-day readmission rate, and occurrence of recurrent colonic diverticulitis.

### Procedure

When diagnosed with uncomplicated diverticulitis, patients were admitted to the hospital and intravenous hydration was initiated while fasting. Treatment of uncomplicated diverticulitis in immunocompetent patients without the use of antibiotics was implemented as the standard-of-care at our hospital. However, administration of antibiotics to patients was decided at the discretion of the treating physician. In the antibiotic treatment group, ceftriaxone 2 g intravenously (IV) once daily and metronidazole 500 mg IV thrice daily were administered; ciprofloxacin 400 mg IV twice daily was substituted in the case of ceftriaxone allergy. A physical examination was performed daily. When symptoms and abdominal tenderness improved, patients were allowed sips of water and then a soft diet. If patients continued to improve after introducing the soft diet, they were discharged from the hospital with an outpatient follow-up visit at 1 to 2 weeks for examining their condition.

In the antibiotic treatment group, antibiotics were only administered during hospital stay, and were not prescribed after discharge.

### Primary and secondary outcomes

The primary outcome of the study was the length of hospital stay. The secondary outcomes were time to the introduction of sips of water and a soft diet, changes in values of laboratory findings (WBC and CRP), the 30-day readmission rate, and the recurrence rate.

### Statistical analyses

Categorized variables were described using numbers with percentages, and continuous variables were described as means ± standard deviations. For comparison between the two treatment groups, categorical variables were analyzed using the χ^2^ or Fisher’s exact tests, and continuous variables were analyzed using the Mann-Whitney or Student’s t-tests. A p-value of less than 0.05 was considered statistically significant. Statistical analysis was performed using SPSS v. 21 (IBM, NY, USA).

The patients were matched between groups using propensity score matching with a 1:1 ratio, using logistic regression with the nearest-neighbor method. Propensity score matching was performed using the R package *MatchIt* (R version 3.2.2) [9]. The variables included in the matching were recorded upon admission, and were as follows: age, sex, height, weight, BMI, heart rate, symptom duration, body temperature, smoking and alcohol use history, presence of underlying disease (hypertension, diabetes, and/or cardiac disease), diverticulum location, initial WBC count, initial segmental neutrophil count, and initial CRP level.

## Results

**Fig 1:**
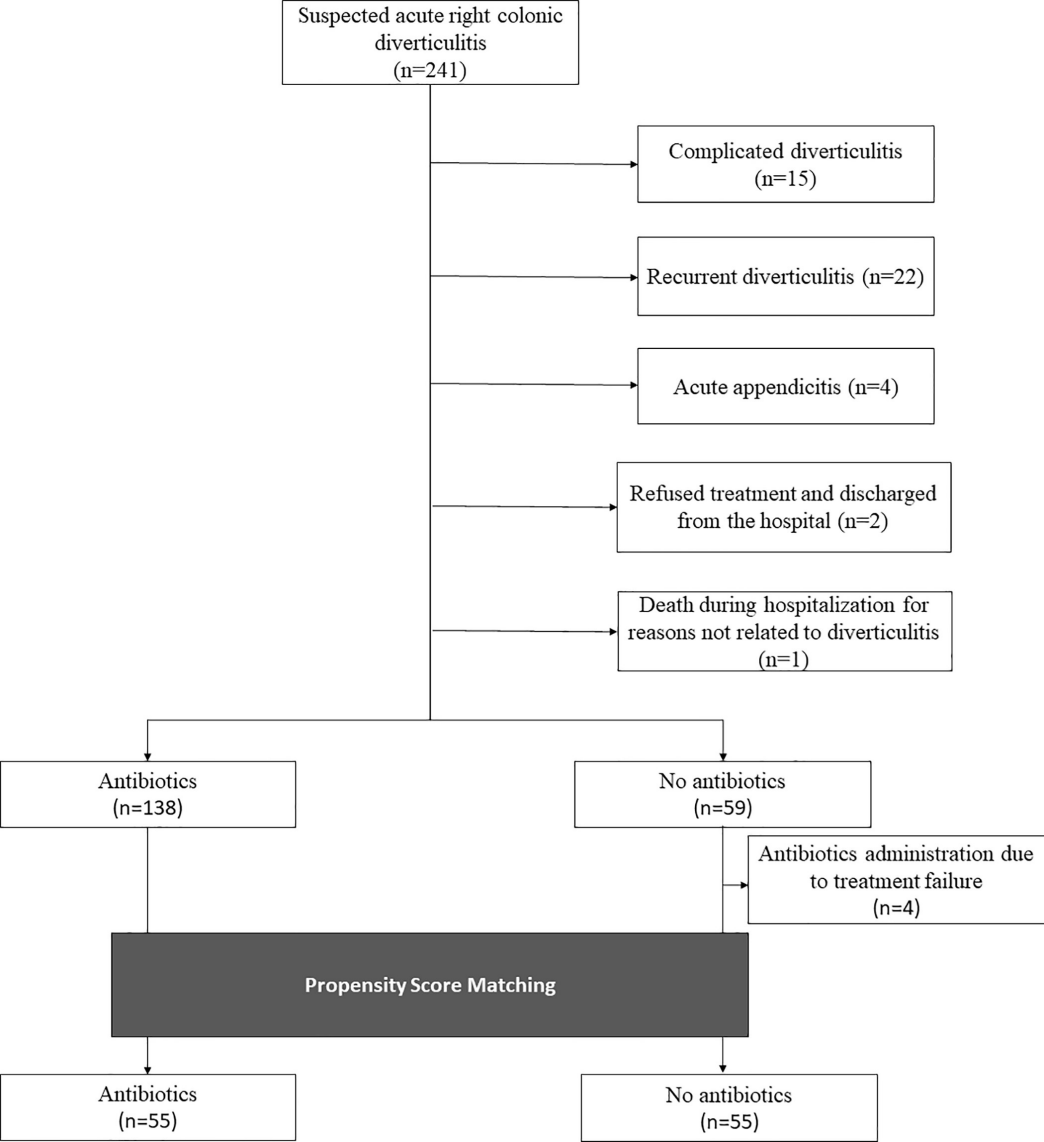
Patient flow diagram.

Among 241 consecutive patients diagnosed with right colonic diverticulitis on CT, 44 were excluded from the analysis owing to complicated diverticulitis (n = 15), recurrent diverticulitis (n = 22), confusion with acute appendicitis (n = 4), refusal of treatment and subsequent discharge from the hospital (n = 2), and death during hospitalization for reasons not related to diverticulitis (n = 1) (Fig 1). In either group, there were no patients with chronic kidney disease, pregnancy, or collagen-vascular disease; none were receiving chronic corticosteroid therapy.

All patients included in this study had stage Ia disease according to the modified Hinchey classification [10]. Among the remaining 197 patients, 138 and 59 were treated with and without antibiotics, respectively. The difference in clinical characteristics between the two groups is shown in Table 1. No variables differed significantly between the two groups, except for body weight (65.2 ± 14.0 vs. 71.1 ± 18.5, p = 0.032) and BMI (23.9 ± 3.8 vs. 25.5 ± 5.4, p = 0.037) (Table 1).

**Table 1:** Patients characteristics before propensity score matching between two treatment groups.

	Antibiotics (n = 138)	No antibiotics (n = 59)	p-value
**Age (years)**	42.8 ± 10.3	39.7 ± 12.0	0.114
**Sex**			0.446
** Male**	62 (44.9%)	30 (50.8%)	
** Female**	76 (55.1%)	29 (49.2%)	
**Height (cm)**	165.1 ± 9.6^a^	166.2 ± 8.4	0.446
**Weight (kg)**	65.2 ± 14.0	71.1 ± 18.5	0.032
**Body mass index (kg/m**^**2**^**)**	23.9 ± 3.8^a^	25.5 ± 5.4	0.037
**Symptom duration (days)**	2.5 ± 7.8	1.8 ± 1.4	
**Social history**			
** Smoking**	49 (35.5%)	26 (44.1%)	0.257
** Alcohol**	55 (39.9%)	31 (52.5%)	0.100
**Medical history**			
** Hypertension**	22 (15.9%)	6 (10.2%)	0.288
** Diabetes**	10 (7.2%)	3 (5.1%)	0.758
** Cardiac disease**	5 (3.6%)	1 (1.7%)	0.671
**Pulse rate (/min)**	78.8 ± 13.2	77.1 ± 13.8	0.412
**Body temperature (°C)**	36.9 ± 0.6	36.8 ± 0.6	0.576
**White blood cell count (10**^**3**^**/μL)**	11.7 ± 3.5	11.3 ± 3.5	0.385
**Segmented neutrophil (%)**	71.7 ± 8.8	69.4 ± 10.4	0.108
**C-reactive protein (mg/dL)**	4.4 ± 4.1	4.3 ± 3.9	0.943
**Diseased segment**			0.811
** Cecum**	39 (28.3%)	16 (27.1%)	
** Ascending colon**	93 (67.4%)	40 (67.8%)	
** Hepatic flexure**	4 (2.9%)	2 (3.4%)	
** Transverse colon**	2 (1.4%)	1 (1.7%)	

^a^ missing date =  1

We performed propensity score matching between the two patient groups. Among those treated without antibiotics, four were excluded because their symptoms were not controlled; 55 patients were therefore included in the matching analysis. After propensity score matching, 55 patients were assigned to each group, with no significant differences in clinical characteristics between the two groups (Table 2).

**Table 2:** Patient characteristics after propensity score matching between two treatment groups.

	Antibiotics (n = 55)	No antibiotics (n = 55)	p-value
**Age (years)**	39.1 ± 12.3	38.5 ± 11.3	0.772
**Sex**			>0.999
** Male**	29 (52.7%)	29 (52.7%)	
** Female**	26 (47.3%)	26 (47.3%)	
**Height (cm)**	166.2 ± 10.3	166.6 ± 8.2	0.818
**Weight (kg)**	67.7 ± 16.7	72.1 ± 18.5	0.198
**Body mass index (kg/m**^**2**^**)**	24.3 ± 4.4	25.8 ± 5.4	0.121
**Symptom duration (days)**			0.727
** 0**	7 (12.7%)	9 (16.4%)	
** 1**	24 (43.6%)	21 (38.2%)	
** 2**	12 (21.8%)	9 (16.4%)	
** 3**	9 (16.4%)	11 (20.0%)	
** 4**	1 (1.8%)	3 (5.5%)	
** 5**	1 (1.8%)	1 (1.8%)	
** 7**	1 (1.8%)	1 (1.8%)	
**Social history**			
** Smoking**	25 (45.5%)	26 (47.3%)	0.848
** Alcohol**	30 (54.5%)	30 (54.5%)	>0.999
**Medical history**			
** Hypertension**	5 (9.1%)	6 (10.9%)	0.751
** Diabetes**	4 (7.3%)	3 (5.5%)	>0.999
** Cardiac disease**	1 (1.8%)	1 (1.8%)	>0.999
**Pulse rate (/min)**	75.1 ± 12.3	77.8 ± 13.9	0.291
**Body temperature (°C)**	36.8 ± 0.5	36.8 ± 0.6	0.661
**White blood cell count (10**^**3**^**/μL)**	11.2 ± 3.9	11.5 ± 3.5	0.688
**Segmented neutrophil (%)**	69.6 ± 9.1	69.5 ± 10.3	0.966
**C-reactive protein (mg/dL)**	3.7 ± 3.9	4.4 ± 3.9	0.337
**Diseased segment**			0.864
** Cecum**	12 (21.8%)	14 (25.5%)	
** Ascending colon**	41 (74.5%)	38 (69.1%)	
** Hepatic flexure**	1 (1.8%)	2 (3.6%)	
** Transverse colon**	1 (1.8%)	1 (1.8%)	

**Fig 2:**
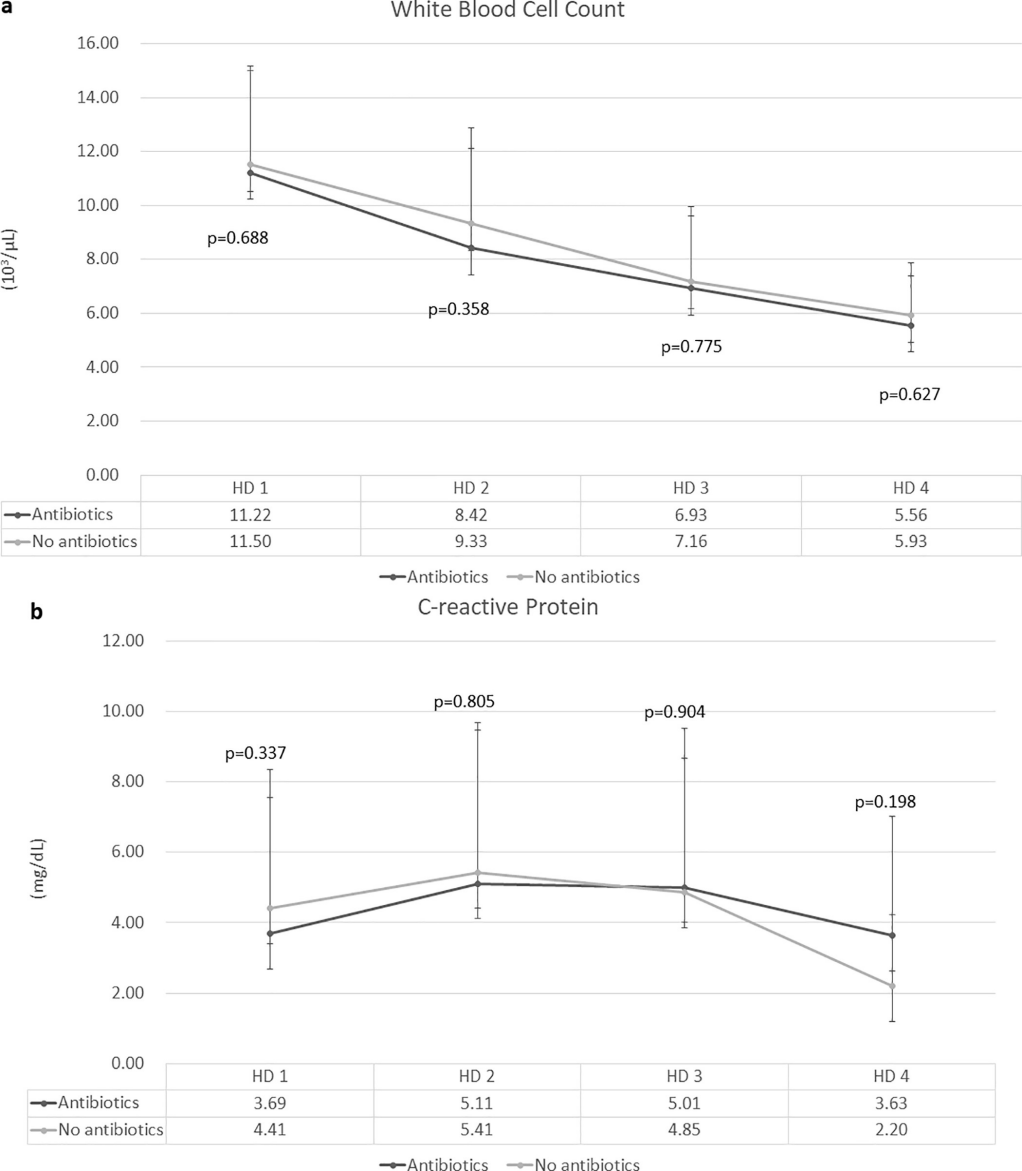
Change in laboratory findings. (a) White blood cell count, (b) C-reactive protein. The white blood cell count showed a tendency to gradually decrease with time (a), but the C-reactive protein level slightly increased on hospital day 2 compared to that on the admission day (hospital day 1) and then decreased thereafter. No factor demonstrated between-group differences over the course of hospital stay. Abbreviations: HD, hospital day.

The primary outcome, i.e., the length of hospital stay, was similar in the antibiotic and no antibiotic groups (3.02 ± 0.95 and 3.11 ± 0.79 days, respectively, p = 0.586). Regarding the secondary outcomes, there were no significant differences between the no antibiotic and antibiotic groups with respect to time to sips of water (2.1 ± 0.7 days vs. 1.8 ± 0.9 days, p = 0.100) and time to soft diet (2.4 ± 0.8 days vs. 2.1 ± 0.9 days, p = 0.125). There was no difference between the two groups with respect to change in values of laboratory findings (WBC and CRP) (Fig 2).

During hospitalization, no patient from either group developed complicated diverticulitis or required surgical intervention; no patients were readmitted 30 days after discharge. A summary of the outcome variables is provided in Table 3.

**Table 3:** Primary and secondary outcomes.

	Antibiotics (n = 55)	No antibiotics (n = 55)	p-value
**Length of hospital stay (days)**			0.584
** 1**	2 (3.6%)	1 (1.8%)	
** 2**	13 (23.6%)	10 (18.2%)	
** 3**	26 (47.3%)	27 (49.1%)	
** 4**	11 (20.0%)	16 (29.1%)	
** 5**	2 (3.6%)	1 (1.8%)	
** 6**	1 (1.8%)	0	
**Time to sips of water (days)**			0.100
** 1**	23 (41.8%)	12 (21.8%)	
** 2**	21 (38.2%)	28 (50.9%)	
** 3**	10 (18.2%)	14 (25.5%)	
** 4**	0	1 (1.8%)	
** 5**	1 (1.8%)	0	
**Time to soft diet (days)**			0.125
** 1**	12 (21.8%)	6 (10.9%)	
** 2**	28 (50.9%)	28 (50.9%)	
** 3**	13 (23.6%)	17 (30.9%)	
** 4**	1 (1.8%)	4 (7.3%)	
** 5**	1 (1.8%)	0	

All the four patients excluded from the propensity matching received antibiotics owing to persistent symptoms during hospitalization. Once antibiotics were initiated, the symptoms in each patient improved, and all of them were discharged from the hospital (Table 4).

**Table 4:** Clinical information regarding four patients with persistent symptoms in the no antibiotic group.

	Patient 1	Patient 2	Patient 3	Patient 4
**Age (years)**	50	53	70	51
**Sex**	Female	Female	Male	Female
**Height (cm)**	148.3	170.6	168	155.8
**Weight (kg)**	45.1	65.9	73	45.55
**Body mass index (kg/m**^**2**^**)**	20.5	22.6	25.9	18.8
**Symptom duration (days)**	1	2	3	3
**Pulse rate (/min)**	68	75	76	53
**Body temperature (°C)**	37.6	36.9	37.1	36.8
**Social history**				
** Smoking**	0	0	0	0
** Alcohol**	1	0	0	0
**Medical history**				
** Hypertension**	0	0	0	0
** Diabetes**	0	0	0	0
** Cardiac disease**	0	0	0	0
**Diseased segment**	Ascending colon	Ascending colon	Cecum	Cecum
**Reason for antibiotics start**	Sustained fever for two days	Sustained pain for three days	Sustained pain for three days	Sustained pain for three days
**Length of stay (days)**	5	5	4	6
**Time to sips of water (days)**	3	3	1	5
**Time to soft diet (days)**	4	3	3	5

During the follow-up period of 229.3 ± 21.9 days for both groups, recurrence after 30 days occurred in 2 (3.6%) patients in the no antibiotic group and in 6 (10.9%) patients in the antibiotic group, without a significant difference between the groups (p = 0.271).

## Discussion

Owing to the lack of evidence regarding clinical guidelines, this study investigated the need for antibiotics in patients with uncomplicated diverticulitis of the right colon. In our propensity-matched analysis of patients treated with or without antibiotics, we did not find any difference between the groups with respect to the length of hospital stay or time to sips of water and introduction of a soft diet. Only 4 (6.7%) patients from the no antibiotic group needed to start taking antibiotics owing to persistent symptoms that subsequently improved without any complications. Finally, there was no difference in 30-day recurrence rates between the two groups.

The necessity of antibiotics in uncomplicated left colonic diverticulitis has been reported in several studies [11–16]; the DIABOLO study was one of the largest multicenter RCTs [13]. In the study, no significant difference was found between the two patient groups with respect to recovery time, which was the primary endpoint (14 days [interquartile range 6–35] for the observation group (n = 262) vs. 12 days [interquartile range 7–30] for the antibiotic treatment group (n = 266), p = 0.151). Furthermore, in the AVOD study (a multicenter RCT), no difference was found in the duration of hospital stay (2.9 ± 1.6 days for the no antibiotic group (n = 309) vs. 2.9 ± 1.9 days for the antibiotic treatment group (n = 314), p = 0.717) [12]. The average age of patients included in these two studies was approximately 57 years, and the frequency of comorbidities was 30%–40%. However, it is known that the common age of onset for right colonic diverticulitis is younger than that for left colonic diverticulitis, and the frequency of comorbidities is low [2–4]. Therefore, some researchers suspect that uncomplicated right colonic diverticulitis can be treated without antibiotics; however, to date, there is insufficient evidence for changing the treatment guidelines.

When we conducted a brief search within several major databases to find literature within the past 5 years, we found one RCT investigating treatment outcomes between a group of Korean patients taking routine antibiotics for uncomplicated right colonic diverticulitis and a group treated without antibiotics [6]. In a total of 125 patients, there was no difference in the length of hospital stay between the groups (p = 0.983), although the mean hospital stay was 5.3 days, which was longer than that in the present study. This raises questions regarding routine antibiotic administration for uncomplicated right colonic diverticulitis. Notably, the overuse of antibiotics may lead to the occurrence of antibiotic-resistant bacteria, as well as various side effects of antibiotic use [17–19]. In particular, some studies have reported that diverticulitis is a type of inflammatory bowel disease rather than colonic microperforation [20, 21].

According to current research, the frequency of antibiotic administration after treatment failure in patients not receiving antibiotics has been reported to be between 2.8% and 4% in cases of uncomplicated left colonic diverticulitis [11, 12, 15]. In each of these studies, the patients recovered without complications after antibiotic treatment. In an RCT on right colonic diverticulitis, the frequency of antibiotic administration after not receiving antibiotics was 3%, which is similar to the findings for left colonic diverticulitis [6]. Compared with these studies, the frequency in our study was slightly higher (6.7%); however, our sample size was smaller than that of other studies. Therefore, future studies with larger sample sizes are needed for comparison. Nevertheless, our study provides evidence that it is safe to administer antibiotics when symptoms or signs do not improve after treatment without antibiotics.

### Limitations

This study had several limitations. First, it had a retrospective design, and was conducted at a single center. However, because we collected patient data on diverticulitis cases, most patients with diverticulitis who visited our hospital during the study period were included. Second, we used a small sample size because the no antibiotic group comprised a lesser number of patients than the antibiotic group before propensity score matching. The minimum required sample size for each group on the basis of the results of our study is approximately 560. Therefore, future studies need to assign more patients to the groups, using a multicenter cohort design. Third, the average follow-up period was relatively short owing to limitations in the patient database. Therefore, future studies are needed to compare the difference in the recurrence rate with a longer follow-up period. Finally, our study focused on diverticulitis in an Asian population, particularly Korean; it is therefore not known whether the results can be generalized to other populations.

## Conclusions

Routine antibiotics did not affect the length of hospital stay in this cohort. Therefore, routine antibiotics may be not required for treating patients with uncomplicated right colonic diverticulitis. It appears to be safe to administer antibiotics in cases of treatment failure after management without antibiotics. However, considering the limitations of our study, a multicenter large-scale prospective study is necessary.
